# Indoor Radon Gas (^222^Rn) Levels in Homes in Aldama, Chihuahua, Mexico and the Risk of Lung Cancer

**DOI:** 10.3390/ijerph15071337

**Published:** 2018-06-26

**Authors:** Carolina Lerma-Treviño, Hector Rubio-Arias, Luis Humberto Colmenero-Sujo, Maria de Lourdes Villalba, Jesus Manuel Ochoa-Rivero

**Affiliations:** 1 College of Animal Science and Ecology of the Autonomous University of Chihuahua, Periferico. R. Almada, km. 1. Chihuahua, Chihuahua C.P. 31453, Mexico; a291034@uach.mx; 2 Technological Institute of Chihuahua II, Ave. de las Industrias 11101, Complejo Industrial Chihuahua, Chihuahua C.P. 31130, Mexico; lh_colmenero@hotmail.com; 3 College of Engineering of the Autonomous University of Chihuahua-Campus II, Circuito Universitario, Chihuahua, Chihuahua C.P. 31125, Mexico; mvillalb@uach.mx; 4 La Campana Experimental Center of the National Research Institute on Forestry, Agriculture and Animal Production (INIFAP), Km. 33.3 Carretera Chihuahua-Ojinaga, Aldama, Chihuahua C.P. 32910, Mexico; ochoa.jesus@inifap.gob.mx

**Keywords:** mining-related disease, natural uranium, Chihuahua, Mexico

## Abstract

Radon (^222^Rn) is an odorless and tasteless gas that is known to cause lung cancer. The objective of this research was to quantify the levels of exposure to radon among people living in an environment rich in uranium (U). Radon concentrations were measured for 3 days in 12 homes in Aldama, Mexico. Homeowners agreed to participate in the study; hence, the sample was non-probabilistic. Radon was measured with a portable AlphaGuard Radon Monitor (Genicron Instruments GmbH), which was placed in a bedroom of each home at a height of 0.74 m. Gas levels were registered in Becquerels (Bq m^−3^), with readings taken every 10 min along with readings of ambient temperature (AT), air pressure (AP), and relative humidity (RH). We found that radon gas levels in Aldama exceed the maximum permissible limits (USA: 148 Bq m^−3^). Levels were higher at night, and were above the maximum permissible level recommended by the International Atomic Energy Agency of the United Nations (<200 Bq m^−3^). Most residents in the area have family histories of lung problems, but it was difficult to establish a strong correlation between ^222^Rn and lung cancer. Federal, state, and municipal governments should take stronger action to reduce the effects of radon gas on communities.

## 1. Introduction

There are high levels of natural uranium (U) in northern Mexico, and consequently high levels of radon gas (^222^Rn). Radon is the product of natural decay of the radioactive elements uranium, thorium (Th), and radium (Ra). Radon has been linked to increased incidence of lung cancer [1,2,3,4,5]. Studies in Costa Rica [6], El Salvador [7], Cuba [8], Kazakhstan [9], India [10], and other countries [11,12] have correlated the presence of radon to lung cancer disease. Radon thus represents a threat to people living in environments where radioactive elements are present [13]. The probability of lung cancer is higher among uranium miners that smoke [9,14,15]. This relationship has been known for a long time: lung cancer was first described in the middle of the 19th century. Craven and Smith [16] reviewed early information on cancer from Lucius Seneca, Georgius Agricola, and Titus Lucretius Carus. Nevertheless, it was not until 1988 that the International Agency for Research on Cancer (IARC) defined radon gas as carcinogenic to humans [17].

It is well-documented that ^222^Rn can be present in low concentrations in outdoor settings [18,19]. However, in enclosed environments, such as unventilated homes, such concentrations of radon can represent a health hazard. Moreover, people living in uranium-rich environments can experience long-term exposure to ^222^Rn, and therefore suffer increased risk of contracting lung cancer. Ground and drinking water can also be affected in radon-rich environments by U, radionuclides, and other contaminants that have a negative effect on human health [20,21]. While there has been very dynamic research into indoor ^222^Rn levels in North America [22], China [23], and Europe [24], this topic has not been adequately studied in Mexico.

The largest uranium deposits in Mexico are in the vicinity of the city of Aldama in Chihuahua State [20]. Radon, which is a product of uranium decay, can migrate from igneous and sedimentary rock rich in uranium to home basements through cracks in walls. The objective of this research was to quantify ^222^Rn levels in homes and estimate exposure levels among people living in an environment rich in natural uranium. This knowledge is important given that contact with radon for most people might be through contamination in their homes. This information can help communities to prevent lung cancer in northern Mexico.

## 2. Materials and Methods

**Figure 1:**
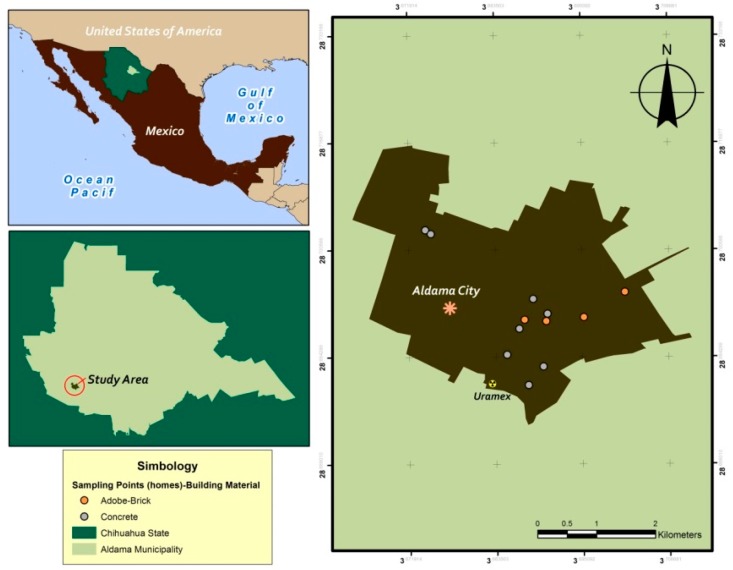
Map showing the Municipality of Aldama, Chihuahua, Mexico.

We selected Aldama for this study because it is well-documented that this area is rich in uranium. The Mexican Uranium Company (URAMEX-Uranio Mexicano) was in fact headquartered in Aldama for over 5 decades before it was shut down. Aldama is located 30 km from the capital city of the State of Chihuahua of the same name (Figure 1) (Lat. 28°50′30.1″ N and Long. 105°55′31.7″ W, at 1700 m above sea level (MASL)). The climate is dry, temperate, and semi-arid, with a maximum temperature of 41.9 °C in summer and a minimum of −7.9 °C in winter. Annual average precipitation is approximately 334 mm [25,26], with rainfall occurring from July to September, with snowfalls sometimes occurring in winter (December–February). The soil derives from igneous and metamorphic rock, and the dominant soils are represented by rhegosols, chernozems, fluvisols, leptosols, and cambisols [27]. Population has been growing at an average annual rate of approximately 2% in the last few decades. The population is currently estimated at approximately 22,010 inhabitants. The total urban area is around 11.83 km^2^.

In winter 2016, 30 homeowners in Aldama were invited by mail to participate in the study and 12 homeowners (40%) agreed to participate (Table 1).

The sample was therefore non-probabilistic, meaning that homeowners in the population did not have equal chances of being selected. Radon concentrations were measured for 3 full days in each home with a portable AlphaGuard radon monitor (Genicron Instruments Model P30 is manufactured by Saphymo GmbH, Heerstraße 149, D-60488, Frankfort, Germany), which was placed in a bedroom of each home at a height of 0.74 m, which is the general height of a person’s head when lying in bed. Radon levels were registered in Becquerels (Bq m^−3^), with readings every 10 min; thus, 6682 measurements were made in total. Ambient temperature (AT), air pressure (AP), and relative humidity (RH) were also measured every 10 min. The types of home construction, in terms of building material and general room distribution, were registered. A specially designed questionnaire with 40 questions was applied to study participants to identify family histories of cancer. In particular, eight questions were related to a family’s background with lung disease (i.e., lung cancer).

An analysis of variance (ANOVA) was applied to the data, looking for differences between house construction types (adobe versus block), and daytime and nighttime measurements. The adobe bricks are made of the soil of the area mixed with water and some sort of organic material, such as straw. All analyses considered a level of significance of 0.01 (α = 0.01). In addition, a correlation coefficient was obtained for AT, AP, RH, and radon concentration. Graphs were prepared as needed to show the concentrations.

## 3. Results

**Figure 2:**
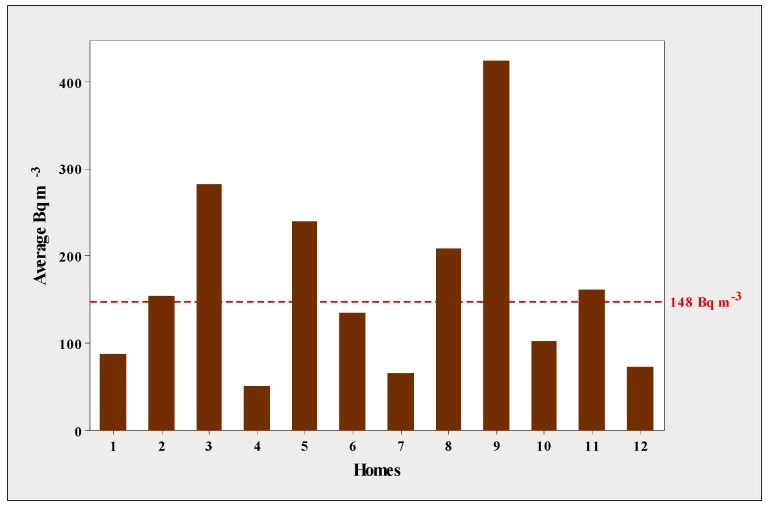
Bar chart of radon levels in 12 homes of Aldama, Chihuahua, Mexico.

**Figure 3:**
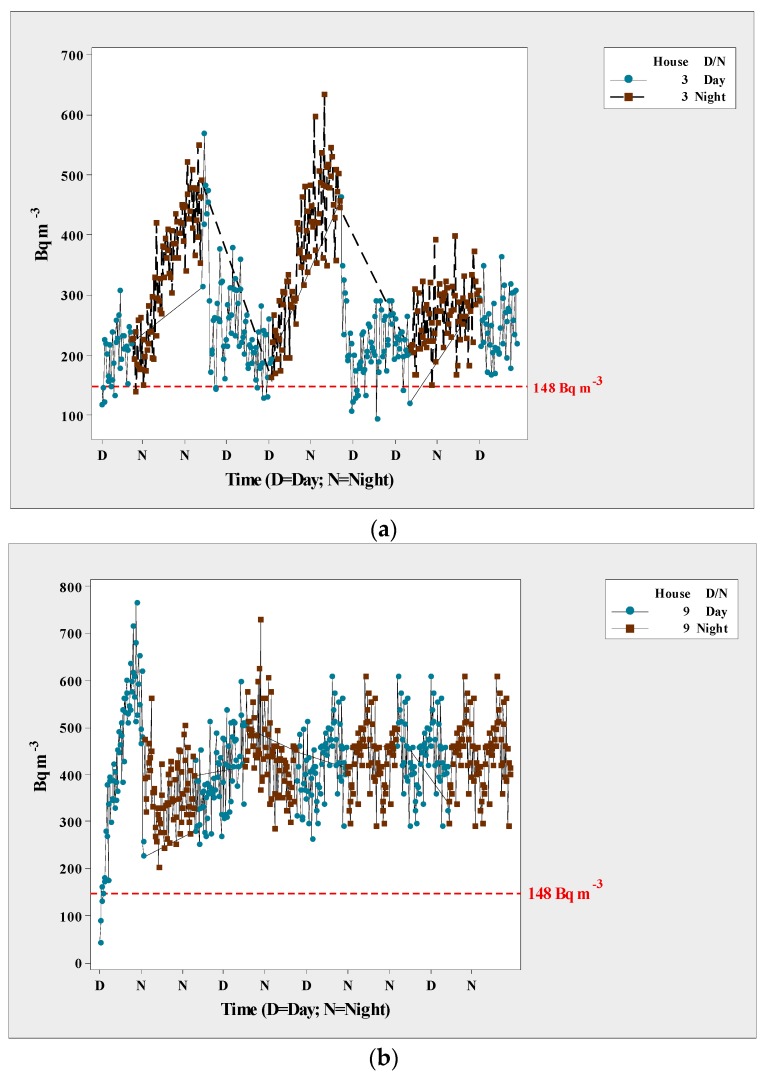
Scatterplot of radon levels in homes 3 (**a**) and 9 (**b**) at Aldama, Chihuahua, Mexico.

The ANOVA detected statistical differences in ^222^Rn levels in the sampled houses (*p* < 0.01). Figure 2 shows the registered levels. Radon levels varied among the homes, with the highest levels in homes 3 and 9, and the lowest in homes 4, 7, and 12. Figure 3 shows the variation in radon concentrations, with the levels found in homes 3 and 9 being well above the maximum safe level of 100 Bq m^−3^ as recommended by the World Health Organization (WHO), and higher than the recommended safe level in the U.S. of 4 pCi L^−1^ (148 Bq m^−3^). It is important to point out that the WHO recommended dropping the exposure levels and suggested to other countries to adopt this reference level.

**Figure 4:**
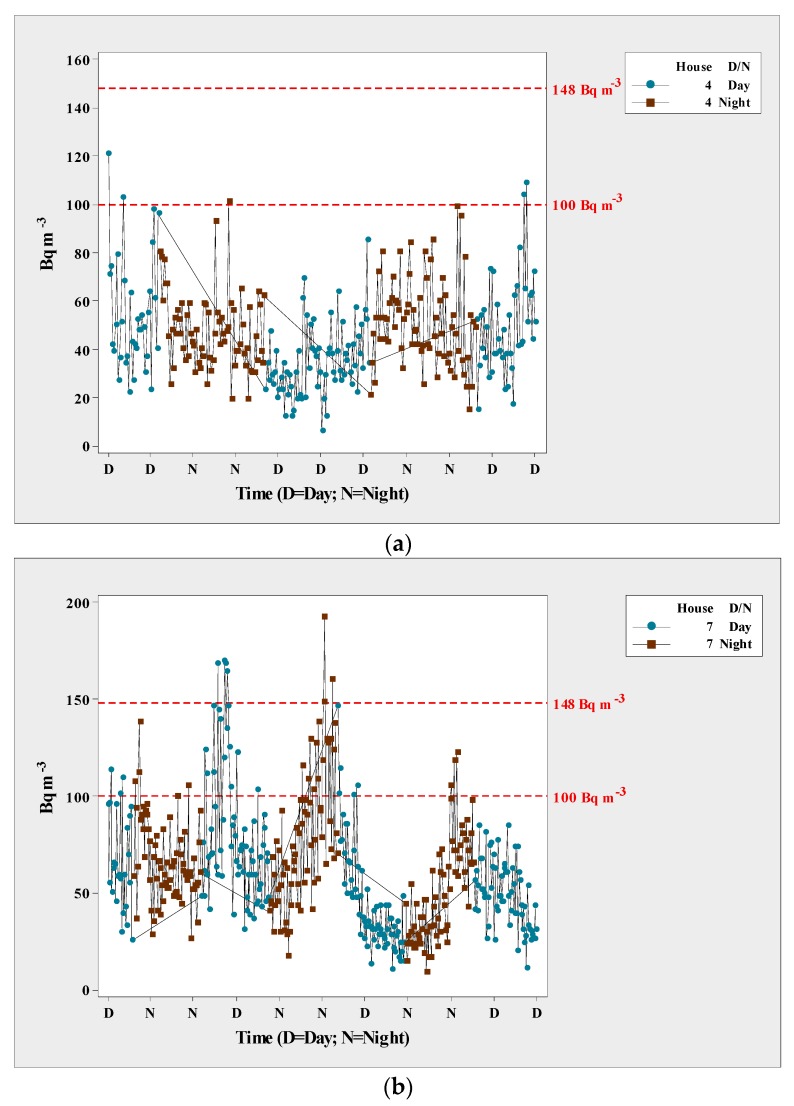
Scatterplot of radon levels in homes 4 (**a**) and 7 (**b**) at Aldama, Chihuahua, Mexico.

It is well-documented that the intensity of radon dissipates in soil, which is why outdoor levels tend to be low even in natural uranium-rich environments. In contrast, indoor environments, such as enclosed houses, can have high levels of radon. Levels can be even higher in poorly ventilated houses, thus increasing the potential harm to their inhabitants because of long-term exposure to the gas. In our study, homes 3, 5, 8, 9, and 11 were built with adobe, which may explain the high concentration of the gas in these homes. On the other hand, homes 1, 2, 4, 6, 7, 10, and 12 were built with block, which is made from concrete and sand (Figure 4). The ANOVA found statistically significant differences in relation to house construction (*p* < 0.01). Figure 3 shows that radon levels were higher at night (peak) than in the daytime. This difference was statistically significant according the ANOVA (*p* < 0.01), and applied to all of the homes. Therefore, the correlation coefficient was significant for temperature.

It is important to point out that humankind receives about 85% of the radiation coming from natural sources and ^222^Rn carries about 50% of that total radiation [28]. It can be hypothesized that people living in Aldama, México will receive a major percentage of radiation coming from ^222^Rn. In fact, five families of this study explained that a relative died of cancer. A national survey in the United States showed a high level of variation in indoor radon levels. Nevertheless, about 26.4% of the homes tested had levels above the recommended maximum of 4.0 pCi L^−1^ [28]. Canada has also tried to identify areas where radon poses a health risk. A survey in Halifax noted that 32% of homes had radon levels above the maximum safe level of 200 Bq m^−3^ as established by Canadian standards. Through interviews and questionnaires, the participants in the study noted that at least one family member had lung cancer or some kind of lung-related illness. In addition, all of them had at least one relative that smoked. It is important to mention that the participants were all middle-class, with similar lifestyles. As noted above, the lack of statistical data from Aldama concerning cancer deaths made it difficult to determine a correlation between radon and the incidence of cancer.

## 4. Discussion

**Figure 5:**
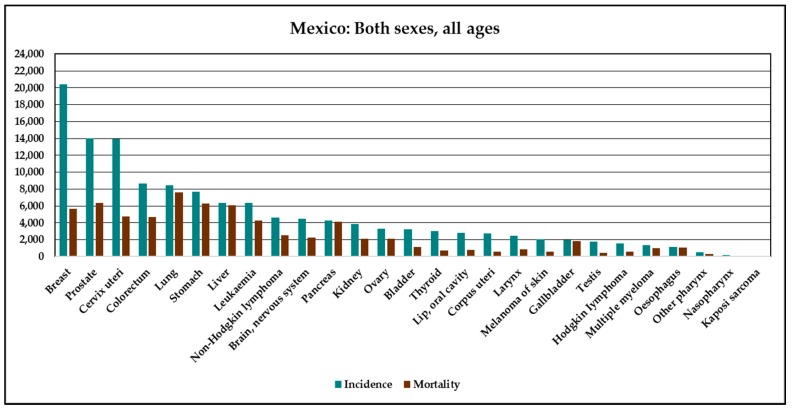
Incidence and mortality of cancer in Mexico (2012); elaborated by the authors with data of GLOBOCAN [17].

The International Agency for Research on Cancer (IARC) has stated that lung cancer has increased in the last decade [29]. In the case of Mexico, lung cancer is presently considered the fifth most common cause of male and female death from cancer after breast, prostate, cervix uteri, and colorectal cancer (Figure 5). Owing to the effect of Mexico’s demographics, the incidence, mortality, and prevalence of lung cancer will increase by about 30%. Notably, the rate of lung cancer is higher among men than among women in Mexico, which is probably related to the higher rate of smoking among males. Despite the importance of lung cancer for human health in Mexico, the most important health institutions, such as the Seguro Popular (a health insurer), the Mexican Institute of Social Security (IMSS), and the Institute of Social Security for State Workers (ISSSTE) do not cover costs for treating lung cancer. According to statistics on mortality rates in Mexico, cancer is the cause of 25% of deaths in northern Mexico, where the State of Chihuahua is located. This is very high considering that death from cancer in the State of Chiapas in southern Mexico accounts for about 3.5% of mortalities.

Our results show that some residents of Aldama might be exposed to certain levels of ^222^Rn in their homes, which potentially represents a risk to human health given that even trace amounts of radon are considered carcinogenic [30]. We understand that, with this data, it is futile to calculate the sampling error and in consequence, an inference can only be drawn for the 12 homeowners and not for the entire population; however, the data is relevant for this community. For instance, a survey carried out in Halifax noted that about 32% of the homes had ^222^Rn levels above Canada’s norm, which specifies a maximum of 200 Bq m^−3^. Exposure is even greater for families living in houses built of adobe, where nighttime levels as high as 600 Bq m^−3^ were registered. It is clear that lowering radon levels in homes will help reduce the incidence of lung cancer among residents of Aldama. In other words, as radon exposure levels decrease, the probability of lung cancer incidence also decreases. Due to the lack of statistical information from Aldama, it is not possible to correlate radon and lung cancer among local inhabitants. It can be hypothesized that most of the radiation that people in Aldama, Mexico are exposed to is in the form of radon.

Cancer statistics in the Municipality of Aldama are even more alarming if we consider that the city is close to the state capital of Chihuahua. Most persons with lung cancer or other kinds of lung disease go to the capital for medical attention, and in case of mortality, their deaths are registered in Chihuahua, which masks the full extent of the effects of radon on people’s health in Aldama. Nevertheless, data analysis from around the world has shown that lung cancer increases with increased presence of radon [24]. Gray et al. [31] reported that 1100 lung cancer deaths in the United Kingdom were related to radon exposure in the home. Catelinois et al. [32] estimated that between 2.2% and 12.4% of lung cancer deaths in France may be attributable to radon gas.

## 5. Conclusions

This study has found that the health of some of Aldama’s inhabitants may be under threat due to radon exposure in their homes. Consequently, epidemiological studies need to be carried out in northern Mexico, in particular in areas with high levels of radon, such as the city of Aldama, Chihuahua. It is also recommended that radon levels be continuously monitored, in particularly in northern Mexico, to obtain data on high-risk areas.

## Figures and Tables

**Table 1:** Location of the homes in Aldama, Chihuahua, Mexico.

Number of Homes	Latitude				Longitude				MASL *	Type of Build
**1**	28°	50′	59.0″	N	105°	56′	15.2″	W	1280	Block
**2**	28°	50′	25.4″	N	105°	54′	25.5″	W	1264	Block
**3**	28°	50′	11.4″	N	105°	54′	48.0″	W	1281	Adobe
**4**	28°	49′	44.3″	N	105°	55′	10.1″	W	1280	Block
**5**	28°	50′	13.3″	N	105°	55′	08.0″	W	1267	Adobe
**6**	28°	49′	50.7″	N	105°	55′	30.1″	W	1270	Block
**7**	28°	50′	56.9″	N	105°	56′	12.1″	W	1293	Block
**8**	28°	50′	21.3″	N	105°	55′	15.9″	W	1274	Adobe
**9**	28°	50′	05.0″	N	105°	55′	02.4″	W	1274	Adobe
**10**	28°	49′	34.2″	N	105°	55′	18.2″	W	1270	Block
**11**	28°	50′	09.2″	N	105°	55′	08.6″	W	1264	Adobe
**12**	28°	50′	09.9″	N	105°	55′	20.5″	W	1277	Block

* MASL: meters above sea level.
